# Tuberculosis of Male Breast: A Rare Benign Entity

**DOI:** 10.7759/cureus.4709

**Published:** 2019-05-21

**Authors:** Kulsoom Fatima, Farah Naz

**Affiliations:** 1 Radiology, Aga Khan University Hospital, Karachi, PAK

**Keywords:** tuberculosis, breast, granulomatous, mastitis

## Abstract

Granulomatous mastitis is a rare benign condition often seen in young lactating females of reproductive age group. Prepubescent males or elderly women may also be the victim of this infectious disease. Primary infection of the breast may occur through skin abrasions, open wounds or through the lactiferous ducts while secondary spread occurs from an infective focus elsewhere in the body via lymphatic or hematogenous routes. We present a case of breast tuberculosis diagnosed in a 62-year-old man at our institution. The patient presented with a palpable painful mass in the left breast with chronic sinus formation and pus discharge for a month with loss of appetite and weight loss. The etiology was unknown. The imaging features were suggestive of tuberculosis. Histopathology was concordant with imaging and showed chronic granulomatous inflammation with necrosis. The patient received oral anti-tuberculosis therapy for six months with no side effects or any further complications. Breast tuberculosis is a rare entity especially in male breast mimicking carcinoma. The mainstay of treatment is antitubercular therapy if imaging and histopathology confirms the diagnosis. Clinical awareness is necessary during diagnostic workup for establishing the correct diagnosis and treatment.

## Introduction

Granulomatous mastitis is a rare benign condition. It is often seen in young lactating females of reproductive age group (20 to 40 years). Prepubescent males or elderly women may also be the victim of this infectious disease [1,2]. The disease usually presents as a lump in the central or upper-outer quadrant of the breast [2]. It is commonly a primary disease where etiology is unknown in >60% of the cases or secondary in the remaining cases. Primary infection of the breast may occur through skin abrasions, open wounds or through the lactiferous ducts [3]. Secondary spread occurs from an infective focus of pulmonary tuberculosis or from other primary sites of tuberculosis via lymphatic or hematogenous routes [4]. In 1972, Kessler and Wolloch first described mammary tuberculosis although the first reported case dates back to 1829 [5, 6]. It usually presents with sinus formation and abscesses. Clinical and radiological features may mimic pyogenic abscess or malignancy [7]. Surgical excision and steroid therapy are the most commonly used treatments [6, 7]. The clinician may misdiagnose breast tuberculosis as either breast carcinoma or abscess before the use of anti-tuberculous therapy [8]. The coexistence of carcinoma and breast tuberculosis adds challenge to the diagnosis. Correct diagnosis of tuberculous mastitis is important as the treatment of differential diseases varies from steroid to unnecessary surgery leading to devastating consequences in patients suffering from breast tuberculosis. In this case report, we describe mammary tuberculosis in a male patient which was clinically suspected to be a malignancy.

## Case presentation

**Figure 1:**
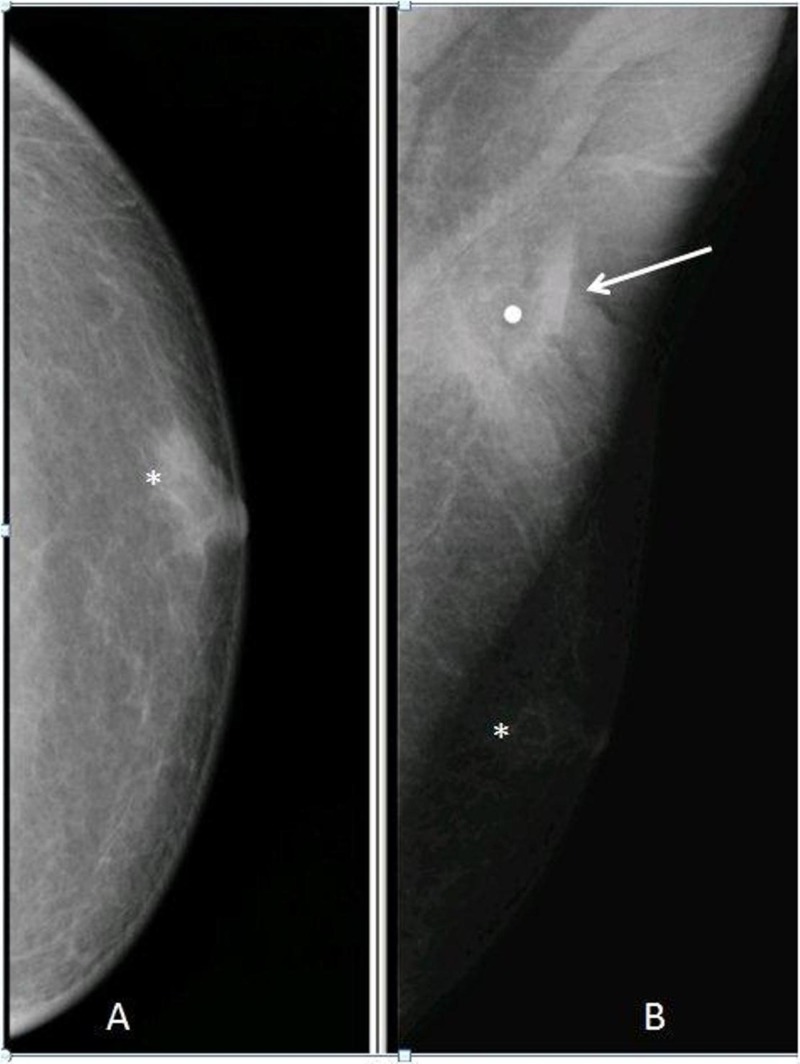
Cranio-caudal (A) and mediolateral oblique view (B) mammogram An irregular soft tissue density is identified on MLO view beneath the metallic marker (arrow) placed at the site of palpable lump in upper half of left breast. Note mild glandular tissue (asterisk) in the retroareolar region. MLO: Mediolateral oblique

Herein we present a case of 62-year-old male who presented to our institution with a painful lump in his left breast with chronic sinus formation and pus discharge. According to the patient, the lump had been noticed for one month. The patient had loss of appetite and weight loss. The patient had a history of a common room with his colleagues during trip to an Asian country. The patient did not have any past or family history of pulmonary or extra-pulmonary tuberculosis. He was neither immune-compromised nor a smoker without any comorbidities. On physical examination, the left breast was tender, and an irregular mass of 3 x 2 cm was felt in upper outer quadrant of the left breast with multiple palpable axillary lymph nodes. Hematological and biochemical parameters were within normal limits, including negative testing for HIV. His initial workup included a mammogram which revealed irregular soft tissue density mass in upper outer quadrant of left breast (Figure 1).

**Figure 2:**
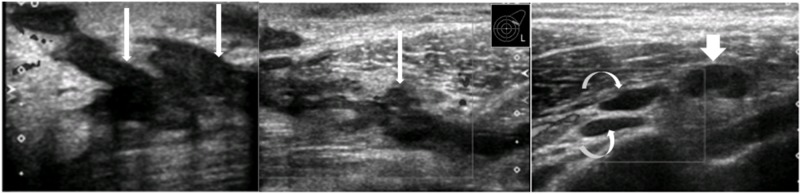
Ultrasound Irregular communicating branching sinus tracts (long white arrows) in the upper outer quadrant of left breast, extending towards axilla (curved white arrows). Enlarged left axillary lymph node (short thick white arrow).

This was followed by ultrasound which showed irregular communicating branching sinus tracts (Figure 2). These were found extending towards left axilla.

**Figure 3:**
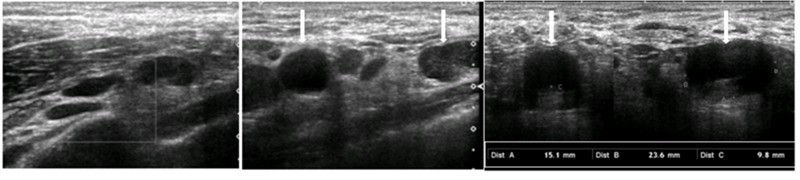
Ultrasound left axilla Enlarged hypoechoic left axillary lymph nodes (white arrows) showing cortical thickening with compressed/absent hilum.

There were multiple enlarged lymph nodes in ipsilateral axilla with thickened cortices. The largest one was measuring 2.3 x 1.5 cm with cortical thickness of 0.98 cm (Figure 3).

A high possibility was raised for communication of these lymph nodes with the sinus tracts leading our diagnosis more towards tuberculosis.

Patient's chest radiograph was normal without any evidence of parenchymal or pleural abnormality. The patient then underwent lymph node biopsy to rule out malignant etiology and to confirm the radiological diagnosis. Histopathology (images not available) revealed multiple epithelioid granulomas and multinucleated Langhan's type giant cells. Acute inflammatory exudate was also noted along with areas of caseous necrosis. There was no evidence of malignancy. It was concluded as chronic granulomatous inflammation with necrosis favoring tuberculosis. Histopathology was concordant with imaging and showed chronic granulomatous inflammation with necrosis. The patient received oral anti-tuberculosis therapy for six months without side effect or complications. Informed consent was taken from the patient before writing the case report.

## Discussion

Tuberculosis of male breast is an extremely rare condition. In a study containing 809 cases of male breast masses, Lilleng et al. did not find a single case of tuberculosis [9]. The common presenting complaint is a unilateral often painful mass with or without sinus formation or ulceration either in upper outer quadrant or central part of breast with palpable lymph nodes in axilla [2]. In our case, the patient was suspected to have infection, but malignancy could not be ruled out due to advanced age. The predisposing factor for breast involvement by tuberculosis was unclear in our patient. This is also reported as unknown in more than 60% of the cases in the published literature [10]. Mammary tuberculosis has three variants, i.e., nodular, disseminated, and sclerosing. The nodular variant is often mistaken for a mass like fibroadenoma or carcinoma. The disseminated variety results in caseation and sinus formation. Our case showed features of both nodular and disseminated forms. Sclerosing tuberculosis is slow growing with absence of suppuration or abscess formation more often seen in older women.

Patients presenting with a breast lump associated with discharging sinuses are easily diagnosed as tuberculosis but need to be differentiated from actinomycosis in which sulphur granules are seen in discharge and by fungal culture. The isolated breast lump without sinuses or suppuration mimics carcinoma as the lump is usually ill-defined, irregular, and occasionally hard. Pain in the tuberculous lump is present more frequently than in carcinoma usually a dull, constant, and non-descriptive ache as seen in our case. Nipple and areola are rarely involved in tuberculosis. Fixation to the skin may be present as part of the inflammatory process, which may resemble an inflammatory carcinoma.

This entity is usually treated by four drug regimens for six months in uncomplicated cases as was done in our case. The patient's symptoms completely resolved. Simple mastectomy may be performed in advanced cases with inflammatory fibrosis causes destruction of the breast.

## Conclusions

Mammary tuberculosis is a rare entity especially in male breast mimicking carcinoma. Diagnosis can be suggested by initial imaging which is finally confirmed on histopathology. The mainstay of treatment is antituberculous therapy. Surgery is only reserved for selected complicated non-responding cases. Clinical awareness is necessary during diagnostic workup for establishing the correct diagnosis and treatment.
